# A Tn*5051*-like *mer*-containing transposon identified in a heavy metal tolerant strain *Achromobacter *sp. AO22

**DOI:** 10.1186/1756-0500-2-38

**Published:** 2009-03-07

**Authors:** Shee Ping Ng, Belinda Davis, Enzo A Palombo, Mrinal Bhave

**Affiliations:** 1 Environment and Biotechnology Centre, Faculty of Life and Social Sciences, Swinburne University of Technology, PO Box 218, Melbourne, Victoria 3122, Australia; 2 School of Molecular Sciences, Victoria University, PO Box 14428, Melbourne, Victoria 8001, Australia

## Abstract

**Background:**

*Achromobacter *sp. AO22 (formerly *Alcaligenes *sp. AO22), a bacterial strain isolated from a lead-contaminated industrial site in Australia, was previously found to be resistant to moderate to high levels of mercury, copper and other heavy metals. However, the nature and location of the genetic basis for mercuric ion resistance in this strain, had not been previously identified.

**Findings:**

*Achromobacter *sp. AO22 contains a functional *mer *operon with all four essential genes (*merRTPA*) and shows >99% DNA sequence identity to that of Tn*501*. The *mer *operon was present on a transposon, designated Tn*AO22*, captured by introducing a broad-host-range IncP plasmid into *Achromobacter *sp. AO22 and subsequently transferring it to *E. coli *recipients. The transposition frequency of Tn*AO22 *was 10^-2 ^to 10^-3 ^per target plasmid transferred. Analysis of Tn*AO22 *sequence revealed it belonged to the Tn*21 *subgroup of the Tn*3 *superfamily of transposons, with the transposition module having >99% identity with Tn*5051 *of a *Pseudomonas putida *strain isolated from a water sample in New York.

**Conclusion:**

Tn*AO22 *is thus a new variant of Tn*5051 *of the Tn3 superfamily and the transposon and its associated mercury resistance system are among the few such systems reported in a soil bacterium. *Achromobacter *sp. AO22 can thus be exploited for applications such as *in situ *mercury bioremediation of contaminated sites, or the mobile unit and *mer *operon could be mobilized to other bacteria for similar purposes.

## Findings

Mercury-resistance encoding *mer *operons have been reported from many bacterial species isolated from diverse environments including pristine soils, ancient permafrost samples, mercury ores as well as contaminated soil or water samples and enterobacteria [1-6]. These are commonly located on mobile genetic elements such as plasmids, transposons or modules of recombinant structures, although some reside chromosomally [6,7]. *mer *transposons frequently belong to the Tn*3 *family where the members are typically flanked by 38 bp inverted repeats (IRs) and contain two genes, *tnpR *and *tnpA*, encoding the enzymes resolvase and transposase, respectively, and a resolution (*res*) site at which site-specific recombination occurs to resolve the cointegrates formed during transposition [8]. Two archetypal transposons of this family, Tn*21*, isolated from plasmid pNR1 from a clinical strain of *Shigella flexneri *from Japan [9], and Tn*501*, from pVS1 from a *Pseudomonas aeruginosa *isolate from Australia [10], have provided in-depth information on the transposition modules as well as functions of individual *mer *genes and regulation of the operon (reviewed in [7,11]). Many of the *mer*-transposons are closely related and share characteristics of the Tn*21 *subgroup of Tn*3*, with genes arranged as *res-tnpR-tnpA, tnpR *and *tnpA *separated by only 2 or 3 bp and transcribed in the same direction, away from *res *[12].

A number of variations on the genetic organisation of *mer *operons from Gram negative bacteria have been reported, but most contain the essential genes *merRTPA *with optional accessory genes (*merB, C, D, E, F, G*) and open reading frames (ORFs). *merR *encodes the transcriptional regulator of the operon, *merT *and *merP *encode a Hg (II) transport system across the cell membrane and *merA *encodes mercuric reductase that reduces the toxic Hg(II) to elemental Hg(0) in the cytoplasm which is released into the environment (reviewed in [7]). The *mer *operons are being prospected intensely for use in developing biosensors for detecting mercury contamination [13,14], and bioremediation/phytoremediation systems [15]. We have previously reported a soil bacterial strain *Achromobacter *sp. AO22 (initially called *Alcaligenes *sp. AO22) from a disused battery-manufacturing site in Melbourne [16], which was tolerant to heavy metals including lead, copper and mercury. This work reports the presence and characterization of a transposon in this strain, with a functional *mer *operon located on it.

### Strain AO22 is identified as *Achromobacter *sp. AO22

The strain AO22 had been previously identified as *Alcaligenes *sp. based solely on metabolic tests [16]. In order to confirm the identity of strain AO22, sequencing of its 16S ribosomal RNA gene was carried out. Amplification of genomic DNA of AO22 with primers fD1 and rP2 based on 16S rDNA of *E. coli *[17] led to a 1,500 bp PCR product. DNA sequencing and blastn analysis indicated that this 1463 bp sequence (GenBank number EU696789) exhibited >99% identity to the corresponding regions of 16S rDNAs of *Alcaligenes faecalis, Achromobacter xylosoxidans *and other *Alcaligenes *spp. and 100% identity with that of *A. faecalis *strain 5659-H (AJ509012). A phylogenetic tree based on the alignment of 16S rDNA of AO22 with that of select type strains of *Achromobacter *spp., *Alcaligenes *spp. and several other β-*Proteobacteria *indicated AO22 and 5659-H belong to the cluster of *Achromobacter *spp. which is relatively distant from *Alcaligenes *spp. (results not shown). Alignment of the AO22 16S sequence with the Ribosomal Database Project  analysis tool also assigned it to the genus *Achromobacter *with 100% confidence. Indeed, the GenBank entry of 5659-H is 16S rDNA of *A. xylosoxidans *subsp. *Xylosoxidans*, as pointed out by Wellinghausen et al. [18]. The AO22 sequence was then aligned with all other type strains of *Achromobacter *spp. and *Alcaligenes faecalis *subsp. *faecalis*, *Bordetella brochiseptica *and *Cupriavidus necator *were included for comparison. From the phylogenetic tree (Additional file 1 Fig. S1), AO22 appears to be most closely related (99.7% identity) to *Achromobacter spanius *and is henceforth designated as *Achromobacter *sp. AO22. As 16S rDNAs of several *Achromobacter *species are >97% identical, DNA-DNA hybridization may be required to further test the relatedness.

### Identification and isolation of Tn*AO22*

*Achromobacter *sp. AO22 was found to carry certain *mer *gene sequences which were more than 90% similar to those in Tn*501*[16]. In order to test whether an active transposon was present in this strain, an approach described by Mindlin et al. [19] was used to mobilize it. This involved a two-step conjugation: (i) introduction of a broad-host-range plasmid from an *E. coli *host to AO22; (ii) determining transposition of the mercury transposon (if present) by mating of AO22 containing this plasmid with an *E. coli *recipient and selecting for transconjugants with linkage of the plasmid marker to mercury resistance. In the first step, a tetracycline (Tc) resistant broad-host-range IncP plasmid pVS520 [20] (Additional file 1: Table S1) was introduced into AO22 by conjugation performed by the spot mating method [21] with modifications. The donor *E. coli *LT104 (pVS520) and recipient (AO22) cultures were grown overnight at 37°C and 30°C respectively in Luria Bertani (LB) broth, with Tc (10 μg ml^-1^) and Hg (HgCl_2_: 0.005 mM) as respective selections. The cultures were diluted 1:100 in fresh LB broth and incubated for a further 5 h with shaking. The donor and recipient cultures were then mixed 1:5, 10 μL aliquots of the mixture spotted on LB agar without selection and incubated for 16–18 h at 37°C. The mixed growth was scraped off the plate, resuspended in 0.85% saline, the suspension serially diluted 10-fold with 0.85% NaCl and 20 μL of each dilution spotted on selective LB agar plates to determine the number of colonies of donor (Tc^r^), recipient (Hg^r^) and transconjugants (Tc^r^Hg^r^), respectively. The conjugation experiment was repeated three times. The transfer frequency of pVS520 was expressed as the number of transconjugants, i.e., AO22 (pVS520) colonies per donor cell, and found to be an average of 1.26 × 10^-6^(SD 7.5 × 10^-7^) from the three independent experiments. The Hg^r^Tc^r ^transconjugant strain, designated AO22 (pVS520), grew better at 37°C and the plasmid in it remained stable after several transfers on selective media. For the transposition experiment, AO22 (pVS520) was subcultured on LB agar containing Hg and Tc daily for three days to ensure maintenance of pVS520, then mated as above with spontaneous rifampicin-resistant mutants of *E. coli *JIR7062 [22] (designated JIR7062R; Additional file 1: Table S1) isolated in-house. The selections used were Tc^r^Hg^r ^for donor, Rif^r ^for recipient (25 μg ml^-1 ^in LB broth, 100 μg ml^-1 ^in LB agar), Tc^r^Rif^r ^for identifying transconjugants with pVS520, and Hg^r^Tc^r^Rif^r ^for identifying transconjugants with pVS520 carrying the potential mercury transposon (tentatively designated Tn*AO22*) transferred onto it. The transposition frequency of Tn*AO22 *was expressed as number of Hg^r^Tc^r^Rif^r ^colonies per Tc^r^Rif^r ^colony, as described by Bogdanova et al. [1]. The transfer frequency of pVS520 from AO22 (pVS520) to JIR7062R averaged 6.9 × 10^-1 ^(± 3.2 × 10^-1^) per donor cell while the frequency of mercury-resistant transconjugants (pVS520 with Tn*AO22*) per pVS520-containing cell (i.e., Tc^r^Rif^r^) was 1.8 × 10^-2 ^(± 1.1 × 10^-2^). This is similar to that for Tn*5044 *[23] and slightly higher than that for other mercury transposons [1,2]. A total of 8 colonies were picked from the *E. coli *transconjugants and restriction analysis of their plasmids revealed identical restriction patterns including an insertion of approximately 8 kb when compared with restriction pattern of pVS520. One of these plasmids (designated pVS520::Tn*AO22*) was used for characterisation of Tn*AO22*.

### Cloning and sequencing of Tn*AO22 *reveal it has all functionally important features and belongs to the Tn*21 *subgroup

**Figure 1:**
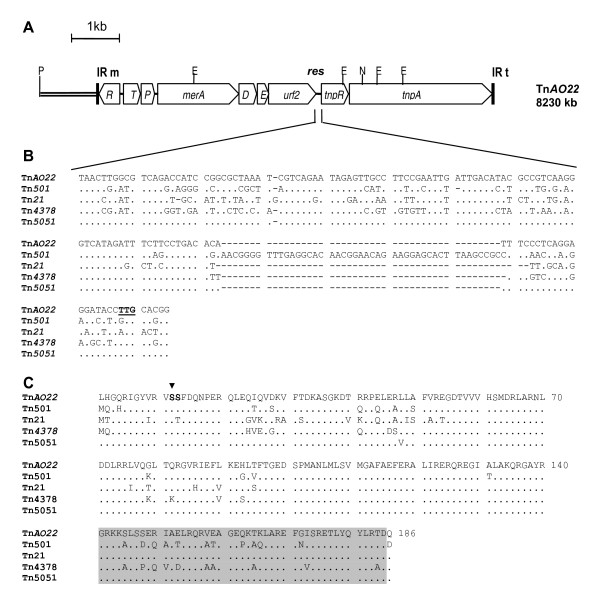
**Genetic organisation of Tn*AO22***. (A) Organisation of the *mer *operon and *tnp *genes. Select restriction sites are shown (E: *Eco*RI; N: *Nco*I; P: *Pst*I). IR: inverted repeats. The solid line between the *Pst*I site and left IR indicates a 1.1 kb section of pSV520 included in the 6.7 kb *Pst*I-*Nco*I fragment of pVS520::Tn*AO22 *cloned into pGEM-T Easy. (B) Comparison of the *res *sites: Tn*501 *from *P. aeruginosa *pVS1 (Z00027), Tn*21 *from *S. flexneri *R100 (NC_002134), Tn*4378 *from *C. metallidurans *CH34 pMOL28 (NC_006525) and Tn*5051 *from *Pseudomonas sp*. (Y17719). Dots indicate nucleotides identical to those of Tn*AO22; *dashes indicate gaps introduced to optimise identity. (C) Comparison of the putative amino acid sequences of resolvase of Tn*AO22 *with those of Tn*501 *(CAA77327), Tn4378 (ABF13038) and Tn*21 *from *S. flexneri *(NP_052901) and Tn*5051 *(CAC14696). Arrow head indicates the presumptive serine involved in recombination. The shaded region indicates the conserved helix-turn-helix motif of resolvases.

**Figure 2:**
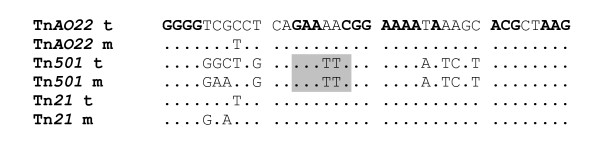
**Comparison of terminal IRs of Tn*AO22 *with IRs of Tn*501 *and Tn*21***. Boldface bases: conserved positions for the efficient recognition by the Tn*21 *transposase; shaded sequences: *Eco*RI sites; t: IRs at the *tnpA *end and 'm' the *mer *end.

A 6.7 kb *Pst*I-*Nco*I fragment of pVS520::Tn*AO22 *was cloned into pGEM^®^-T Easy vector (Promega Australia) for sequencing purposes. This fragment was sequenced initially using the vector-based primers T7 (5'-GTAATACGACTCAGGGC-3') and SP6 (5'-TTTAGGTGACACAGAATC-3'). As data was generated, a further section of Tn*AO22 *was amplified using pVS520::Tn*AO22 *as template and the primers AO22-F (5'-GACGAATACGGGCAGCGG-3') designed 70 bp upstream of the *Nco*I site and VS520-R (5'-GGCGGCGGTGTGGAAGC-3') designed 100 bp into sequence of pVS520. PCR products were purified and sequenced as above, using the primers used for PCR and additional primers designed based on the emerging sequence data. DNA sequences were assembled and analyzed using the Bioedit Alignment Editor v.7.0.9 . The most closely related sequences were found using the Basic Local Alignment Search Tool (BLAST) program , multiple alignments were performed with CLUSTALW  and phylogenetic and evolutionary analyses conducted using MEGA version 4 . The sequence data indicated that the 6.7 kb *Pst*I-*Nco*I fragment of pVS520::Tn*AO22 *contained a 1.1 kb section of pVS520, followed by one end of the putative transposon, a putative *mer *operon, a *tnpR *gene, and part of *tnpA *(Fig. 1A). A primer designed approximately 70 bp upstream of the *Nco*I site using these data, in combination with a primer designed approximately 100 bp into pVS520, gave a 2.8 kb PCR product from pVS520::*TnAO22 *templates isolated from *E. coli *cells. The sequences of the 6.7 kb *Pst*I-*Nco*I fragment and this PCR product were assembled and showed Tn*AO22 *had a length of 8230 bp (GenBank number EU696790). It was inserted 173 bp downstream of the truncated Tn*1 *in pVS520 (in pVS520::Tn*AO22*), equivalent to position 10614 of RP1 (BN000925), and had resulted in 5 bp duplications (TCTAT) of target sequence in the flanking region of pVS520 (data not shown), the latter being a characteristic of Tn*3 *family [12]. Tn*AO22 *was bounded by 38 bp imperfect IRs differing by only 1 bp (Fig. 2), the IR adjacent to *mer *operon being identical to that of Tn*21 *at the *tnpA *end. The IRs were highly similar to those of the ancestral Tn*501 *except its *Eco*RI sites and contained conserved sequences recognized by the Tn*21 *transposase [12]. The Tn*AO22 *insertion site in pVS520 (equivalent to a region between Tn*1 *and oriV in RP1 or its derivatives) appears to be a hot spot for insertions, as reported for several Tn*5041*-type elements [24]. Nine ORFs were identified within Tn*AO22*, the first seven closest to the IR from insertion point containing sequences homologous to the *mer *operon, including a *merR *that terminated within the adjacent IR and *merTPADEurf2 *transcribed divergently, and the two other ORFs being similar to *tnpR *and *tnpA *genes and separated from the *mer *ORFs by a 131 bp sequence similar to the *res *site (Fig. 1A).

The DNA sequence of the *res-tnpR *region of Tn*AO22 *had highest identity (>99%) to this region of Tn*5051 *isolated from a *P. putida *strain in a water sample in New York [19] (Table 1). For optimum alignment with other related transposons, gaps needed to be introduced, the most notable one being a 45 bp gap between the 3' end of *res *and start codon of *tnpR *of Tn*AO22 *compared to Tn*501 *and other sequences (Fig. 1B). This extra sequence in Tn*501 *is suggested to be the remainder of a transposon belonging to the Tn*5041*/κ branch of Tn*3 *[23]. The putative 186 amino acid TnpR of Tn*AO22*, when aligned with its closest relatives, revealed only one difference with Tn*5051 *(V48L) and conservation of the invariant serine and the helix-turn-helix DNA-binding motif (Fig. 1C).

**Table 1:** DNA sequence identity between Tn*AO22 *and its closest relatives.

**Genome region**		Tn*AO22*	Tn*501*	Tn*21*	Tn*4378*	Tn*5051*
Tn*AO22*	*mer*^*a*^	100	99.8	73.4	99.8	na^b^
	*res-tnpR*	100	73.1	79.6	81.3	99.5
	*tnpA*	100	70.0	90.0	92.3	99.2
Tn*501*	*mer*		100	73.4	99.8	na^b^
	*res-tnpR*		100	67.5	75.2	72.9
	*tnpA*		100	68.9	69.6	69.9
Tn*21*	*mer*			100	73.4	na^b^
	*res-tnpR*			100	74.3	79.4
	*tnpA*			100	90.5	89.9
Tn4378	*mer*				100	na^b^
	*res-tnpR*				100	81.1
	*tnpA*				100	91.9
Tn*5051*	*mer*					na^b^
	*res-tnpR*					100
	*tnpA*					100

^*a*^*mer *includes *merRTPAD *and *merE*; ^b^% identity with Tn*5051 *was not calculated for this region, as only 339 bp sequence data is available for Tn*5051*. Accession numbers: Tn*501 *from *Pseudomonas aeruginosa *plasmid pVS1 (Z00027), Tn*21 *from *Shigella flexneri *plasmid R100 (pNR1) (NC_002134), Tn*4378 *from *Ralstonia metallidurans *CH34 plasmid pMOL28 (NC_006525) and Tn*5051 *from *Pseudomonas putida *(Y17719).

**Figure 3:**
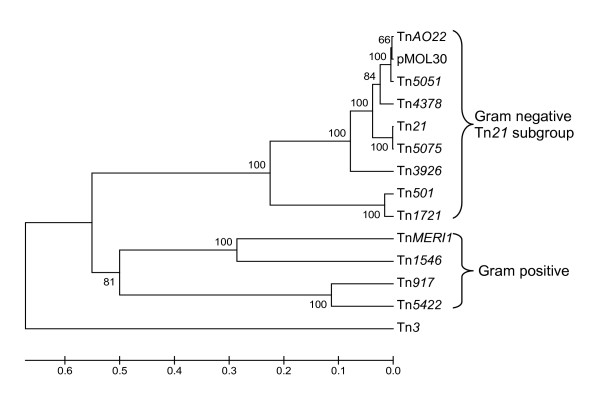
**Neighbour-joining distance dendogram of the amino acid sequences of transposases of the Tn*3 *family**. Bootstrap percentages (100 replicates) are shown to the left of the node being considered. Genbank accession numbers: pMOL30, YP_145631; Tn*5051*, CAC14696; Tn*4378*, YP_161722; Tn*5075*, AF457211; Tn*3926*, X14236; Tn*MERI1*, AB022308; Tn*1721*, X61367; Tn*917*, M11180; Tn*5422*, L28104; Tn*1546*, M97297; Tn*3*, V00613.

The DNA sequence identities of Tn*AO22 tnpA *compared to its close relatives varied between 70.0% and 99.2% (Table 1). The start codon of the putative TnpA was 2 bp after termination of TnpR (data not shown), compared to 3 bp in Tn*501*, and it terminated within an IR. The putative TnpA is 988 amino acids long and differs from the 459 residues available for TnpA of Tn*5051 *at 5 positions. Alignment of the amino acid sequence of Tn*AO22 *TnpA with 13 selected Tn*3 *transposases (Additional file 1 Fig. S2) and the dendrogram (Fig. 3) confirmed that Tn*AO22 *was closest to the Tn*21 *subgroup of Gram negative transposons, separated from Tn*3 *and the cluster of transposons in Gram positive bacteria.

### Tn*AO22 *houses a mer operon that is very similar to that on Tn*501*

The *mer *operon of Tn*AO22 *had the classical structure *merRTPADE*, with >99% identity to Tn*501 *at DNA level including the length and sequences of intergenic spacers, and lacked the *merC *gene noted in Tn*21 *[11]. As in Tn*501*, the putative operator/promoter regions and transcription start sites of *merR *and *merTPAD *were divergent and the potential binding site of the regulator MerR occurred between the start codons of MerR and MerT. The putative mercuric reductase MerA of Tn*AO22 *was 561 amino acids long, and comparison of its putative N-terminal and C-terminal sequences to MerA of other organisms (Additional file 1 Fig. S3) showed the two conserved pairs of cysteines considered responsible for binding and catalytic reduction of Hg(II) to Hg(0). Downstream of *merD *were sequences similar to *orf1 *and *orf2 *of Tn*501*. The putative protein encoded by *orf1 *was homologous to the 78 amino acid protein now known as MerE and suggested to have a role in Hg(II) transport similar to MerT [11], while *orf2 *encodes a 329 amino acid homologue of the diguanylate phosphodiesterases with the conserved EAL domain thought to be involved in prokaryotic signal transduction pathways [25].

With some exceptions, many mercury resistance determinants are located on plasmids. However, no plasmid could be isolated from AO22 despite numerous attempts using various volumes of cell culture and alternative protocols, e.g., standard alkaline lysis, the method of Kado and Liu [26] and two commercial kits. This, however, does not rule out the possibility of a megaplasmid in AO22 on which Tn*AO22 *is located, as it is often difficult to detect such plasmids using common methods as well as to totally exclude them from chromosomal DNA preparations. The genomic DNA of AO22 showed positive hybridisation with a *tnpR *probe (data not shown). Further work would be required using approaches such as quantitative PCR or Southern hybridisations to test whether there is more than one mercury transposon in AO22, located on its main chromosome and/or plasmid, as in case of *Cupriavidus metallidurans *CH34 [27];. The fact that AO22 was receptive to introduction of a broad-host-range IncP plasmid indicates any resident plasmid(s) is (are) unlikely to belong to this incompatibility group. The fact that the *mer *operon of Tn*AO22 *is functional (and not a relic) can be inferred from conferral of mercuric ion resistance to the *E. coli *host in conjugation experiments. Lack of *merB *genes suggests narrow spectrum Hg resistance; confirmed on plates containing organomercurials (Davis and Bhave, unpublished). Other mercury-resistant Gram-positive and Gram-negative bacterial genera have been isolated from the same site [16]; it would be interesting to explore whether transposons similar to Tn*AO22 *are also present in these strains.

Based on sequence identities and *res-tnpR-tnpA *gene organisation, Tn*AO22 *appears to belong to the Tn*21 *branch of the Tn*3 *subgroup of transposable elements [12]. The structures of the *mer *operon and transposition modules of Tn*AO22 *suggest it is a recombinant transposon, probably a variant of Tn*5051*. The putative resolvases (TnpR) of both transposons have TTG as the possible start codon; though rare, this start codon has been reported among prokaryotes, notably for *lacA *in the *E. coli lac *operon [28]. The *mer *operon of Tn*5051 *is nearly identical to that of Tn*501*, and based on the proposed evolution of Tn*501 *[19], Tn*AO22 *and Tn*5051 *probably share an ancestor with Tn*501 *from which the *mer *operon originated. Very closely related *mer *transposons are reported from diverse strains and geographical locations, e.g., at least 10 variants of Tn*5053 *worldwide [6]. Tn*AO22 *appears to be a new variant of Tn*5051 *and may be involved in horizontal transfer of mercury resistance, possibly giving the host a selective advantage in contaminated sites such as the one *Achromobacter *sp. AO22 was isolated from. *mer*-mediated removal of mercury from sewage and industrial effluent has been described [15,29]. *Achromobacter *sp. AO22 is one of the few soil bacterial species to contain *mer *genes and is thus well suited for *in situ *bioremediation or conjugal transfer of mercury resistance to indigenous soil community, as shown for enhanced degradation of organic contaminants [30].

## Abbreviations

IR: inverted repeat; Tn: transposon; Hg^r^: mercury resistant; Tc^r^: tetracycline resistant.

## Competing interests

The authors declare that they have no competing interests.

## Authors' contributions

SPN carried out the microbiological and molecular genetic studies, sequence alignments and drafted the manuscript. BD participated in the sequencing. EAP and MB conceived of the study, participated in its design and coordination and helped to draft the manuscript. All authors read and approved the final manuscript.

## Supplementary Material

Additional File 1 **Supplelmentary Table S1 and Figures S1–S3**. **Table S1**. Bacterial strains and plasmids; **Figure S1**. Neighbour-joining distance dendogram of the 16S rDNA sequences of the genus *Achromobacter *and related species and **Figure S2: **Multiple alignments of the putative transposase of Tn*AO22 *with those of selected transposons from Gram positive and Gram negative bacteria. **Figure S3**. Comparison of the amino acid sequences of the N-terminal (A) and C-terminal (B) sections of the putative MerA of Tn*AO22 *with MerA of selected bacteria. Click here for file

## References

[B1] Bogdanova E, Minakhin L, Bass I, Volodin A, Hobman JL, Nikiforov V (2001). Class II broad-spectrum mercury resistance transposons in Gram-positive bacteria from natural environments. Res Microbiol.

[B2] Essa AM, Julian DJ, Kidd SP, Brown NL, Hobman JL (2003). Mercury resistance determinants related to Tn*21*, Tn*1696*, and Tn*5053 *in enterobacteria from the preantibiotic era. Antimicrob Agents Chemother.

[B3] Holt RJ, Bruce KD, Strike P (1999). Conservation of transposon structures in soil bacteria. FEMS Microbiol Ecol.

[B4] Huang C-C, Narita M, Yamagata T, Itoh Y, Endo G (1999). Structure analysis of a class II transposon encoding the mercury resistance of the Gram-positive bacterium *Bacillus megaterium *MB1, a strain isolated from Minamata Bay, Japan. Gene.

[B5] Kholodii G, Mindlin S, Petrova M, Minakhina S (2003). Tn*5060 *from the Siberian permafrost is most closely related to the ancestor of Tn*21 *prior to integron acquisition. FEMS Microbiol Lett.

[B6] Mindlin SZ, Bass IA, Bogdanova ES, Gorlenko ZM, Kalyaeva ES, Petrova MA, Nikiforov VG (2002). Horizontal transfer of mercury resistance genes in environmental bacterial populations. Mol Biol.

[B7] Barkay T, Miller SM, Summers AO (2003). Bacterial mercury resistance from atoms to ecosystems. FEMS Microbiol Rev.

[B8] Sherratt D, Berg DE, Howe MM (1989). Tn*3 *and related transposable elements: site-specific recombination and transposition. Mobile DNA.

[B9] Nakaya R, Nakamura A, Murata Y (1960). Resistance transfer agents in *Shigella*. Biochem Biophys Res Commun.

[B10] Stanisich VA, Bennett PM, Richmond MH (1977). Characterization of a translocation unit encoding resistance to mercuric ions that occurs on a nonconjugative plasmid in *Pseudomonas aeruginosa*. J Bacteriol.

[B11] Liebert CA, Hall RM, Summers AO (1999). Transposon Tn*21*, flagship of the floating genome. Microbiol Mol Biol Rev.

[B12] Grinsted J, de la Cruz F, Schmitt R (1990). The Tn*21 *subgroup of bacterial transposable elements. Plasmid.

[B13] Bontidean I, Mortari A, Leth S, Brown NL, Karlson U, Larsen MM, Vangronsveld J, Corbisier P, Csoregi E (2004). Biosensors for detection of mercury in contaminated soils. Environ Pollut.

[B14] Hansen LH, Sørensen SJ (2000). Versatile biosensor vectors for detection and quantification of mercury. FEMS Microbiol Lett.

[B15] Wagner-Dobler I (2003). Pilot plant for bioremediation of mercury-containing industrial wastewater. Appl MicrobiolBiotechnol.

[B16] Trajanovska S, Britz ML, Bhave M (1997). Detection of heavy metal ion resistance genes in Gram-positive and Gram-negative bacteria isolated from a lead-contaminated site. Biodegradation.

[B17] Weisburg WG, Barns SM, Pelletier DA, Lane DJ (1991). 16S ribosomal DNA amplification for phylogenetic study. J Bacteriol.

[B18] Wellinghausen N, Wirths B, Poppert S (2006). Fluorescence in situ hybridization for rapid identification of *Achromobacter xylosoxidans *and *Alcaligenes faecalis *recovered from cystic fibrosis patients. J Clin Microbiol.

[B19] Mindlin S, Kholodii G, Gorlenko Z, Minakhina S, Minakhin L, Kalyaeva E, Kopteva A, Petrova M, Yurieva O, Nikiforov V (2001). Mercury resistance transposons of gram-negative environmental bacteria and their classification. Res Microbiol.

[B20] Palombo EA, Yusoff K, Stanisich VA, Krishnapillai V, Willetts NS (1989). Cloning and genetic analysis of *tra *cistrons of the Tra 2/Tra 3 region of plasmid RP1. Plasmid.

[B21] Fong ST, Stanisich VA (1989). Location and characterization of two functions on RP1 that inhibit the fertility of the IncW plasmid R388. J Gen Microbiol.

[B22] Schneider K, Beck CF (1986). Promoter-probe vectors for the analysis of divergently arranged promoters. Gene.

[B23] Kholodii G, Yurieva O, Mindlin S, Gorlenko Z, Rybochkin V, Nikiforov V (2000). Tn*5044*, a novel Tn*3 *family transposon coding for temperature-sensitive mercury resistance. Res Microbiol.

[B24] Kholodii G, Yurieva OV, Gorlenko Z, Mindlin SZ, Bass IA, Lomovskaya OL, Kopteva AV, Nikiforov VG (1997). Tn*5041*: a chimeric mercury resistance transposon closely related to the toluene degradative transposon Tn*4651*. Microbiology.

[B25] Galperin MY, Nikolskaya AN, Koonin EV (2001). Novel domains of the prokaryotic two-component signal transduction systems. FEMS Microbiol Lett.

[B26] Kado CI, Liu ST (1981). Rapid procedure for detection and isolation of large and small plasmids. J Bacteriol.

[B27] Mergeay M, Monchy S, Vallaeys T, Auquier V, Benotmane A, Bertin P, Taghavi S, Dunn J, Lelie D van der, Wattiez R (2003). *Ralstonia metallidurans*, a bacterium specifically adapted to toxic metals: towards a catalogue of metal-responsive genes. FEMS Microbiol Rev.

[B28] Hediger MA, Johnson DF, Nierlich DP, Zabin I (1985). DNA sequence of the lactose operon: the *lacA *gene and the transcriptional termination region. Proc Natl Acad Sci USA.

[B29] Hansen CL, Zwolinski G, Martin D, Williams JW (1984). Bacterial removal of mercury from sewage. Biotechnol and Bioengin.

[B30] Newby DT, Josephson KL, Pepper IL (2000). Detection and characterization of plasmid pJP4 transfer to indigenous soil bacteria. Appl Environ Microbiol.

