# Supplementary material for: A Tn5051-like mer-containing transposon identified in a heavy metal tolerant strain Achromobacter sp. AO22
Source: BMC Res Notes. 2009 Mar 7;2:38. doi: 10.1186/1756-0500-2-38 (PMC2663772; doi:10.1186/1756-0500-2-38)
Supplement: Additional File 1 — Supplelmentary Table S1 and Figures S1–S3. Table S1. Bacterial strains and plasmids; Figure S1. Neighbour-joining distance dendogram of the 16S rDNA sequences of the genus Achromobacter and related species and Figure S2: Multiple alignments of the putative transposase of TnAO22 with those of selected transposons from Gram positive and Gram negative bacteria. Figure S3. Comparison of the amino acid sequences of the N-terminal (A) and C-terminal (B) sections of the putative MerA of TnAO22 with MerA of selected bacteria. [file 1756-0500-2-38-S1.doc]

**ADDITIONAL FILE 1**

## Supplementary Table S1 and Figures S1-S3

**Table S1**. **Bacterial strains and plasmids**

| **Bacterial strain/**  **plasmid** | **Relevant characteristics* and**  **application in the current study** | **Reference** |
| --- | --- | --- |
| *Achromobacter* sp. AO22 | Wild type, Hg^r^ | [16] |
| *Achromobacter* sp. AO22 (pVS520) | AO22 harbouring pVS520, Hg^r^, Tc^r^ | This study |
| *E. coli* LT104 (pVS520) | LT104, a derivative of UB281 (*pro*, *met*, Nal^r^) with a chromosomal Tn5 element, harbouring pVS520 | [29] |
| *E. coli* JIR7062R (CB454) | Rif^r^ mutant of JIR7062, F^-^, ∆*lacZ*^-^, *lacY*^+^, *galK*, *rpsL*, *thi*, *recA56*, conjugation recipient | [30] |
| pVS520 | IncP, Tc^r^, derivative of RP1; Mobilisation of Tn*AO22* | [29] |
| pVS520::Tn*AO22* | Tc^r^, Hg^r^; Sequencing of Tn*AO22* | This study |

*Selective agent concentrations (µg ml^-1^): tetracycline (Tc, 10); rifampicin (Rif, 25 in LB, 100 in LB agar); Hg (HgCl_2_; 0.005mM).

**Figure S1**

**Figure S1.** **Neighbour-joining distance dendogram of the 16S rDNA sequences of the genus *Achromobacter* and related species.** Bootstrap percentages (1000 replicates) are shown to the left of the nodes. Numbers in parentheses: GenBank accession numbers for 16S rDNA sequences. The scale bar indicates genetic distance: 0.01= 1% dissimilarity. A section of the 16S rRNA gene was amplified from total genomic DNA of strain AO22 using the universal primers fD1 (5’-GAGTTTGATCCTGGCTCAG- 3’) and rP2 (5’-ACGGCTACCTTGTTACGACTT-3’) based on *E. coli* positions 8–27 and 1512-1492 respectively [17]. PCR was performed using an initial denaturation (94°C, 5 minutes), then 35 cycles of denaturation (94°C, 45 seconds), annealing (55°C, 45 seconds) and extension (72°C, 1 minute), then a final extension (72°C, 10 minutes). The PCR products were purified with the Perfect Prep Gel Clean-up kit (Eppendorf, Germany), then sequenced using ABI BigDye Terminator reagent v3.1 chemistry and as per the instructions of the Australian Genome Research Facility (AGRF; St Lucia, Australia; [http://www.agrf.org.au](http://www.agrf.org.au/)) and subjected to separation using a 3730*xl* DNA Analyzer (Applied Biosystems) at AGRF.

**Figure S2**

**Figure S2: Multiple alignments of the putative transposase of Tn*AO22* with those of selected transposons from Gram positive and Gram negative bacteria.** Accession numbers: Tn*501*, Z00027; *Cupriavidus metallidurans* CH34 plasmid pMOL30, CP000354; Tn*21*, NP_052901; Tn*5075*, AF457211; Tn*3926*, X14236; Tn*1721*, X61367; Tn*4378*, NC_006525; Tn*5051* Y17719; Tn*MERI1*, AB022308; Tn*917*, M11180; Tn*5422*, L28104; Tn*1546*, M97297 and Tn*3*, V00613.

**Figure S3**

**Figure S3.** **Comparison of** **the amino acid sequences of the N-terminal (A) and C-terminal (B) sections of the putative MerA of Tn*AO22* with MerA of selected bacteria.**

*P. aeruginosa* Tn*501* (CAA77323); *S. flexneri* Tn*21* (NP_052885); *C. metallidurans* CH34 Tn*4378* (Y_161727); *Alcaligenes sp*. pMER610 (P94188); *Pseudomonas sp.* Tn*5041* (CAA67451); *E. coli* (AAN87562); *E. faecium* (AAR10425); *S. aureus* pI258 (AAA98245); *B. cereus* RC607 (AAA83977). The two pairs of Cys responsible for binding and catalytic reduction of Hg(II) to Hg(0) are highlighted.
